# AON-based degradation of c.151C>T mutant *COCH* transcripts associated with dominantly inherited hearing impairment DFNA9

**DOI:** 10.1016/j.omtn.2021.02.033

**Published:** 2021-03-01

**Authors:** Erik de Vrieze, Jorge Cañas Martín, Jolien Peijnenborg, Aniek Martens, Jaap Oostrik, Simone van den Heuvel, Kornelia Neveling, Ronald Pennings, Hannie Kremer, Erwin van Wijk

**Affiliations:** 1 Department of Otorhinolaryngology, Radboud University Medical Center, 6525 GA Nijmegen, the Netherlands; 2 Donders Institute for Brain, Cognition, and Behaviour, Radboud University Medical Center, 6525 GA Nijmegen, the Netherlands; 3 Department of Human Genetics, Radboud University Medical Center, 6525 GA Nijmegen, the Netherlands; 4 Radboud Institute for Health Sciences, Radboud University Medical Center, 6525 GA Nijmegen, the Netherlands

**Keywords:** antisense oligonucleotides, deafness, hearing loss, genetic therapy, DFNA9, *COCH*, gapmer, RNase H1

## Abstract

The c.151C>T founder mutation in *COCH* is a frequent cause of late-onset, dominantly inherited hearing impairment and vestibular dysfunction (DFNA9) in the Dutch/Belgian population. The initial clinical symptoms only manifest between the 3rd and 5th decade of life, which leaves ample time for therapeutic intervention. The dominant inheritance pattern and established non-haploinsufficiency disease mechanism indicate that suppressing translation of mutant *COCH* transcripts has high therapeutic potential. Single-molecule real-time (SMRT) sequencing resulted in the identification of 11 variants with a low population frequency (<10%) that are specific to the c.151C>T mutant *COCH* allele. Proof of concept was obtained that gapmer antisense oligonucleotides (AONs), directed against the c.151C>T mutation or mutant allele-specific intronic variants, are able to induce mutant *COCH* transcript degradation when delivered to transgenic cells expressing *COCH* minigenes. The most potent AON, directed against the c.151C>T mutation, was able to induce a 60% decrease in mutant *COCH* transcripts without affecting wild-type *COCH* transcript levels. Allele specificity decreased when increasing concentrations of AON were delivered to the cells. With the proven safety of AONs in humans, and rapid advancements in inner ear drug delivery, our *in vitro* studies indicate that AONs offer a promising treatment modality for DFNA9.

## Graphical abstract

**Figure:**
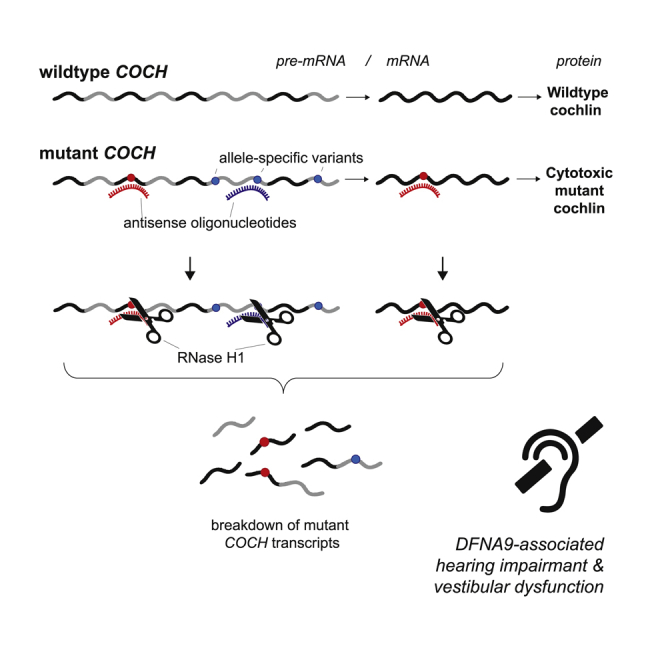

## Teaser

The c.151C>T founder mutation in *COCH* is a frequent cause of late-onset, dominantly inherited hearing impairment and vestibular dysfunction (DFNA9). We designed and *in vitro* validated gapmer AONs that are able to target the mutant *COCH* transcript for degradation. Our results describe the first step toward an AON-based treatment for DFNA9.

## Introduction

DFNA9, caused by mutations in the *COCH* gene, is a relatively common form of dominantly inherited highly progressive hearing loss and vestibular dysfunction. It is characterized by adult-onset hearing loss, leading to complete deafness by the age of 50–70 years.^1^^,^^2^ With progression of the disease, speech perception and conversation become severely limited. DFNA9 patients furthermore suffer from balance problems, which severely hamper their daily activities. Overall, the problems associated with DFNA9 have a severe impact on the quality of life of patients and their relatives and friends.^3^

The *COCH* gene is located on chromosome 14, and encodes cochlin, a protein that consists of 550 amino acids. Cochlin is predicted to contain a signal peptide, an LCCL (limulus factor C, Cochlin, and late gestation lung protein Lgl1) domain, two short intervening domains, and two vWFA (von Willebrand factor A) domains. Cochlin is expressed in fibrocytes of the spiral ligament and spiral limbus, where it has been reported to assist in structural support and sound processing, and in the vestibular fibrocytes that are important in the maintenance of balance.^4^ Proteolytic cleavage of cochlin, between the LCCL domain and the more C-terminal vWFA domains, results in a 16-kDa LCCL domain-containing peptide that is secreted and has been shown to play a role in innate immunity in the cochlea.^5^ The vWFA domain-containing cochlin fragments are presumed to be extracellular matrix proteins, as cochlin vWFA2 was found to interact with collagens *in vitro*, and cochlin is a major component of the cochlear extracellular matrix.^1^^,^^6^

The c.151C>T (p.Pro51Ser) founder mutation, affecting the LCCL domain, appears to be the most prevalent mutation in *COCH*, as it underlies hearing loss in >1,000 Dutch and Belgian individuals.^7^^,^^8^ Histopathology of a temporal bone from a p.Pro51Ser DFNA9 patient revealed significant loss and degeneration of fibrocytes in the cochlea.^1^ Overexpression of murine cochlin containing the ortholog of the p.Pro51Ser variant in cultured cells previously revealed that this mutation results in the formation of cytotoxic cochlin dimers and oligomers that sequester wild-type cochlin.^9^ While the proteolytic cleavage of cochlin was shown to be reduced by the p.Pro51Ser variant and abolished by several other DFNA9-associated variants,^9^ the potential contribution of decreased proteolytic cleavage to DFNA9 pathology requires further investigation.

All available data indicate that DFNA9 results from a gain-of-function and/or a dominant-negative disease mechanism, rather than from haploinsufficiency. Downregulation of the mutant allele, thereby alleviating the inner ear from the burden caused by the formation of cytotoxic cochlin dimers, therefore has high therapeutic potential. The lack of auditory and vestibular phenotypes in mice carrying a heterozygous protein-truncating mutation in *Coch*,^10^ and in heterozygous family members of patients with early-onset hearing impairment due to homozygous protein-truncating mutations in *COCH*,^11^ illustrate that sufficient functional cochlin proteins can be produced from a single healthy *COCH* allele. We speculate that a timely intervention might even prevent hearing impairment altogether.

Antisense oligonucleotides (AONs) with DNA-like properties can be employed to target (pre-)mRNA molecules for degradation by the RNase H1 endonuclease.^12^^,^^13^ Chemical modifications can be introduced in the 5′ and 3′ flanking nucleotides to increase stability and nuclease resistance, while maintaining a central gap region of oligo-deoxynucleotides to bind to the target RNA and thereby activate RNase H1.^12^ These AONs are named gapmers, and their ability to specifically target mutant alleles for degradation has shown great promise in treatment strategies for non-haploinsufficiency disorders such as Huntington’s disease.^14^^,^^15^

For a successful application of AON therapy for non-haploinsufficiency disorders such as DFNA9, it is of major importance that the designed AONs only target the mutant (pre-)mRNA, and not the wild-type (pre-)mRNA, for degradation. As the options to design allele-discriminating AONs based on a single nucleotide difference are limited, we used single-molecule real-time (SMRT) sequencing to identify additional allele-discriminating variants that can be exploited for AON design. This resulted in the identification of 11 variants with a low population frequency (<10%) that are specific to the c.151C>T mutant *COCH* haplotype. Our results show that both the c.151C>T mutation in *COCH* and low-frequency variants in *cis* with the DFNA9 mutation can be used to specifically target mutant *COCH* transcripts for degradation by RNase H1. Lead molecule c.151C>T AON-E appears to be the most promising molecule for further preclinical investigation. As this AON targets the DFNA9-causing mutation, future clinical application is not limited by the potential presence of the target on the patient’s wild-type allele.

## Results

### Identification of therapeutic targets

**Figure 1:**
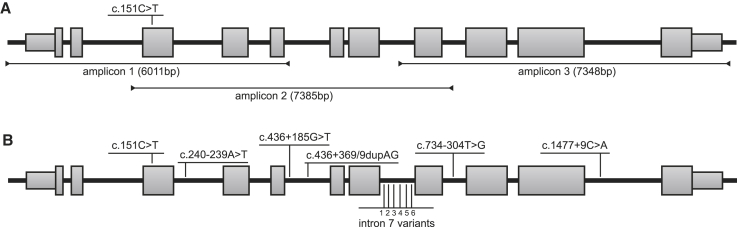
*COCH* haplotype analysis (A) Overview of the amplicons used to determine the haplotype-specific variants on the c.151C>T mutant *COCH* allele. Amplicon length is indicated in base pairs (bp) between brackets. (B) Variants with a low population frequency (<10%) on the c.151C>T mutant haplotype. The six identified variants in intron 7 are 1: c.629+1186T>C; 2: c.629+1779delC; 3: c.629+1807delA; 4: c.629+1809A>C; 5: c.629+1812A>T; 6: c.630-208A>C. Intron-exon structure of transcript NM_001135058.1 is depicted. The c.151C>T variant, causative for DFNA9, is indicated in bold.

In order to develop a mutant-allele-specific therapy for DFNA9, reliable discrimination between the mutant and the wild-type allele is of vital importance. However, the single-nucleotide changes in *COCH* underlying most cases of DFNA9 restrict the design of allele-discriminating therapies. In search of additional variants that can be exploited to improve discrimination between the c.151C>T mutant and wild-type *COCH* allele, we subjected the genomic *COCH* sequence of three DFNA9 patients to long-read SMRT sequencing. We amplified the *COCH* gene in three fragments that contain overlapping single-nucleotide polymorphisms (SNPs) (c.151C>T and c.734-304T>G) to aid haplotype assembly (Figure 1A). The identified variants are annotated on transcript NM_001135058.1, which does not contain the extended second coding exon. To identify targetable allele-specific variants that potentially allow for the treatment of the majority of the Dutch/Belgian DFNA9 patients, we filtered the variants in *cis* with the c.151C>T mutation for a population frequency below 20%. This resulted in the identification of 11 deep-intronic variants that are specific for the c.151C>T mutant *COCH* allele and have population frequencies between 5% and 10% (Figure 1B; Table 1). The identified variants provide additional targets for the development of a mutant allele-specific genetic therapy. The identified variants were validated using Sanger sequencing and confirmed to segregate with the c.151C>T mutation in *COCH* in two branches of Dutch DFNA9 families (Figure S1).

**Table 1:** Identified low-frequency variants on the c.151C>T *COCH* haplotype

Location	SNP identifier	Nucleotide change (HGVS)	Amino acid change	Frequency (percentage) gnomAD European non-Finnish
e4	rs28938175	c.151C>T	Pro51Ser	T: 0.0032
i4	rs143609554	c.240-239A>T		T: 5.4
i6	rs7140538	c.436+185G>T		T: 5.5
i6	rs10701465	c.436+368_436+369dupAG		dupAG: 5.5
i8	rs186627205	c.629+1186T>C		C: 5.4
i8	rs200080665	c.629+1779delC		delC: 5.4
i8	rs368638521	c.629+1807delA		delA: 5.9^a^
i8	rs554238963	c.629+1809A>C		C: 9.9^a^
i8	rs184635675	c.629+1812A>T		T: 5.4
i8	rs2295128	c.630-208A>C		C: 5.3
i9	rs28362773	c.734-304T>G		G: 7.2
i11	rs17097458	c.1477+9C>A		A: 5.4

HGVS, Human Genome Variant Society.

a No data in gnomAD; frequency data from dbSNP 153.

### Design and *in silico* analysis of AONs

**Figure 2:**
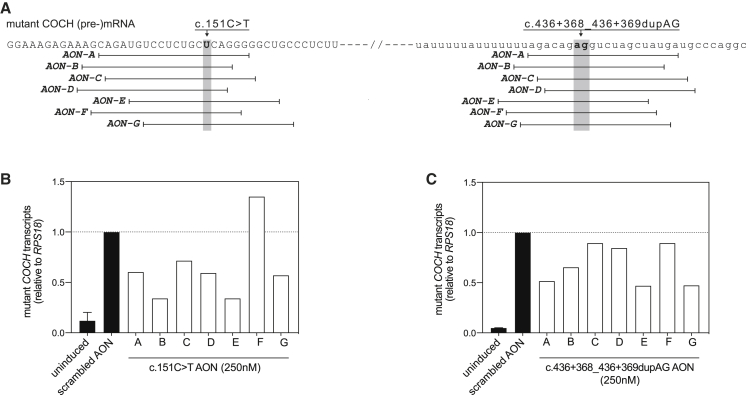
Design and identification of candidate AONs directed against the c.151C>T mutation and the in *cis* intronic variant c.436+368_436+369dupAG (A) Graphical representation of AON-RNA binding positions on the c.151C>T mutant *COCH* transcript. Coding sequences are shown in capitals, intronic sequences in lower case. AON sequences are provided in Table S1. (B) Degradation of mutant *COCH* transcripts by AONs (250 nM end concentration in the medium), directed against the c.151C>T mutation, in mutant *COCH*-expressing transgenic cells. Six out of the seven AONs were able to lower the levels of mutant *COCH* transcripts at 24 h post transfection as compared to cells transfected with a scrambled control AON. (C) Degradation of mutant *COCH* transcripts by different AONs (250 nM end concentration in the medium), directed against the c.436+368_436+369dupAG variant on the mutant *COCH* transcript, in mutant *COCH*-expressing transgenic cells. Four out of the seven AONs showed an obvious decrease in mutant *COCH* transcript levels at 24 h post transfection as compared to cells transfected with a scrambled control AON. Uninduced and scrambled controls are displayed as the average of three biological replicates. Single transfections are used for the screening of on-target AONs. Data are displayed as the fold change compared to scrambled control AON-treated cells and normalized for the expression of *RPS18*.

We selected the c.151C>T founder mutation and the intronic, mutant-allele-specific variant c.436+368_436+369dupAG as targets for AON-based therapy. In contrast with the identified single-nucleotide changes or deletions, the c.436+368_436+369dupAG variant is the only multi-nucleotide variant that is specific to the mutant allele. Based on this, we hypothesized that AONs directed against this variant can provide the highest allele specificity. To design AONs, we combined the criteria that are commonly used to design splice-switching AONs with the previously established notion that RNase H1-dependent AONs require a series of nucleotides with DNA-like properties in their central region.^16^, ^17^, ^18^ All possible AONs were investigated for thermodynamic properties *in silico*, with particular attention to the difference in binding affinity between the mutant and wild-type *COCH* mRNA. Targeting regions of all AONs used in this study are shown in Figure 2A. Note that the difference in binding affinity between the mutant and wild-type *COCH* mRNA was predicted to be larger for the AONs directed against the dupAG variant (c.436+368_436+369dupAG) compared to those directed against the single nucleotide substation (c.151C>T) (Table S1). The recognition of RNA/DNA duplexes by RNase H1 relates to the nature of the carbohydrate moiety in the AON backbone (2′-ribose versus 2′-deoxyribose).^16^ Therefore, AONs were either comprised completely of phosphorothioate (PS)-linked DNA bases, or of a central “gap” region of PS-DNA bases flanked by wings of 2′-O-methyl-RNA bases (gapmers). The gapmer design is particularly suitable for clinical application, as the nuclease-resistant 2′-modified ribonucleotides provide an increased binding affinity and half-life time.^12^^,^^19^^,^^20^

### Establishing stable transgenic cell lines expressing wild-type or c.151C>T *COCH* minigenes

The *COCH* expression levels in patient-derived primary fibroblast and Epstein-Barr virus-transformed lymphoblastoid cells are too low to reliably determine the effect of RNase H1-dependent AONs. Therefore, we used the Flp-In system to generate two stable transgenic T-REx 293 cell lines, expressing either a mutant (including three deep-intronic allele-discriminating variants; Figure 1) or a wild-type *COCH* minigene construct under the control of a tetracycline-dependent promotor. The minigene constructs span the genomic *COCH* sequence between the transcription initiation site and the last complete nucleotide triplet of exon 7 (Figure S2A). For both alleles, several clones were expanded and investigated for inducible *COCH* expression (Figure S2B). Correct pre-mRNA splicing of both wild-type and mutant minigene *COCH* exons 1–7 was confirmed with RT-PCR (Figure S2C). In order to reliably quantify mutant and wild-type *COCH* transcript levels, we used a custom TaqMan assay (Applied Biosystems), in which different fluorophores are coupled to probes specific for either the mutant or the wild-type transcript.

### RNase H1-dependent AONs target mutant *COCH* transcripts for degradation

As the *COCH* gene is continuously expressed in the human cochlea, we opted for an experimental design in which *COCH* transcription remains active. To induce *COCH* transcription, seeded cells were treated overnight with tetracycline (0.25 μg/mL). Next morning, culture medium was replaced by fresh tetracycline-containing medium, and cells were transfected with the AONs. For the initial screening of AONs, we transfected AONs (n = 1) to a final concentration in the culture medium of 250 nM. This dose was selected based on the work of Naessens et al.,^21^ who showed a 50%–70% decrease in target transcripts in an overexpression-based cell model to identified candidate AONs for the future treatment of *NR2E3*-associated retinitis pigmentosa. Six (out of seven) AONs directed against the c.151C>T mutation (Figure 2B) and four (out of seven) AONs directed against the dupAG variant (Figure 2C) were able to decrease the level of mutant *COCH* transcripts as compared to a scrambled control AON.

**Figure 3:**
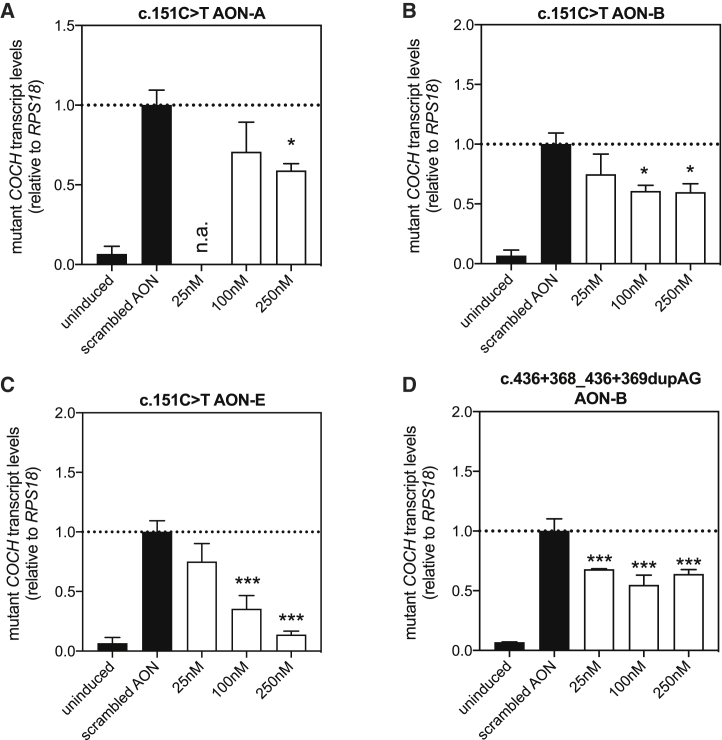
Identified candidate AONs induce a significant decrease in mutant *COCH* transcript levels (A–D) To confirm the effect of previously identified candidate AONs, c.151C>T AON-A (A), c.151C>T AON-B (B), c151C>T AON-E (C), and c.436+368_436+369dupAG AON-B (D) were investigated at two different doses. (A) c.151C>T AON-A results in significant decrease in mutant *COCH* transcripts at 250 nM, but not at 100 nM. (B) c.151C>T AON-B was able to induce a significant decrease in mutant *COCH* transcripts at both 100 nM and 250 nM, but no differences between the two doses were observed. (C) c.151C>T AON-E decreased the level of mutant *COCH* transcripts in a statistically significant and dose-dependent manner. At a concentration of 250 nM, the number of *COCH* transcripts was reduced to 20% of those in cells treated with a scrambled control AON. (D) Transfection of c.436+368_436+369dupAG AON-B resulted in a significant decrease of mutant *COCH* transcripts, without statistically relevant differences between the two concentrations. All four AONs had a gapmer design with wings of 2′-O-methyl-RNA bases flanking the central PS-DNA core. AONs were transfected at a dose leading to the indicated concentration in the well and investigated for their effect on transcript levels 24 h after transfection. Data are expressed as mean ± SD of 3 replicate transfections, normalized to the expression of RPS18 and displayed as the fold change compared to scrambled control AON-treated cells. ∗p < 0.05, ∗∗p < 0.01, ∗∗∗p < 0.001, ∗∗∗∗p < 0.0001, one-way ANOVA with Tukey’s post-test.

Three of the most effective AONs directed against the c.151C>T mutation, and one AON directed against the dupAG variant, were analyzed in more detail using different concentrations of gapmer AONs and multiple technical replications (Figure 3). c.151C>T AON-A was able to induce a significant decrease in mutant *COCH* transcripts at a dose of 250 nM (p = 0.02, Tukey’s multiple comparison test), but not at 100 nM (Figure 3A). While AON-B showed a stronger effect in comparison to AON-A in the initial screening, the effect sizes of AON-A and -B were very similar in this replication experiment (Figure 3B). A significant decrease of mutant *COCH* transcripts was found at 100 nM and 250 nM (p < 0.0012, Tukey’s multiple comparison test). The third AON directed against the c.151C>T mutation that was investigated in more detail, AON-E, did show a typical dose-dependent decrease in mutant *COCH* transcript levels. At 100 nM, the level of mutant *COCH* transcripts was approximately half of the number of transcripts detected in cells treated with a scrambled control AON (p < 0.0002, Tukey’s multiple comparison test). Mutant *COCH* transcript levels were even further decreased in cells transfected with 250 nM of AON-E (p < 0.0001, Tukey’s multiple comparison test). While on average the AONs directed against the dupAG variants appeared slightly less effective in the initial AON screen, transfection of mutant *COCH*-minigene-expressing cells with dupAG AON-B resulted in a significant decrease in mutant *COCH* transcripts at the three concentrations tested (Figure 3D; p < 0.0009, Tukey’s multiple comparison test). The highest effect size appears already to be achieved at 25 nM. The maximum effect size of dupAG AON-B was similar to the effect observed for c.151C>T AON-A and -B, but it was much less when compared to c.151C>T AON-E.

**Figure 4:**
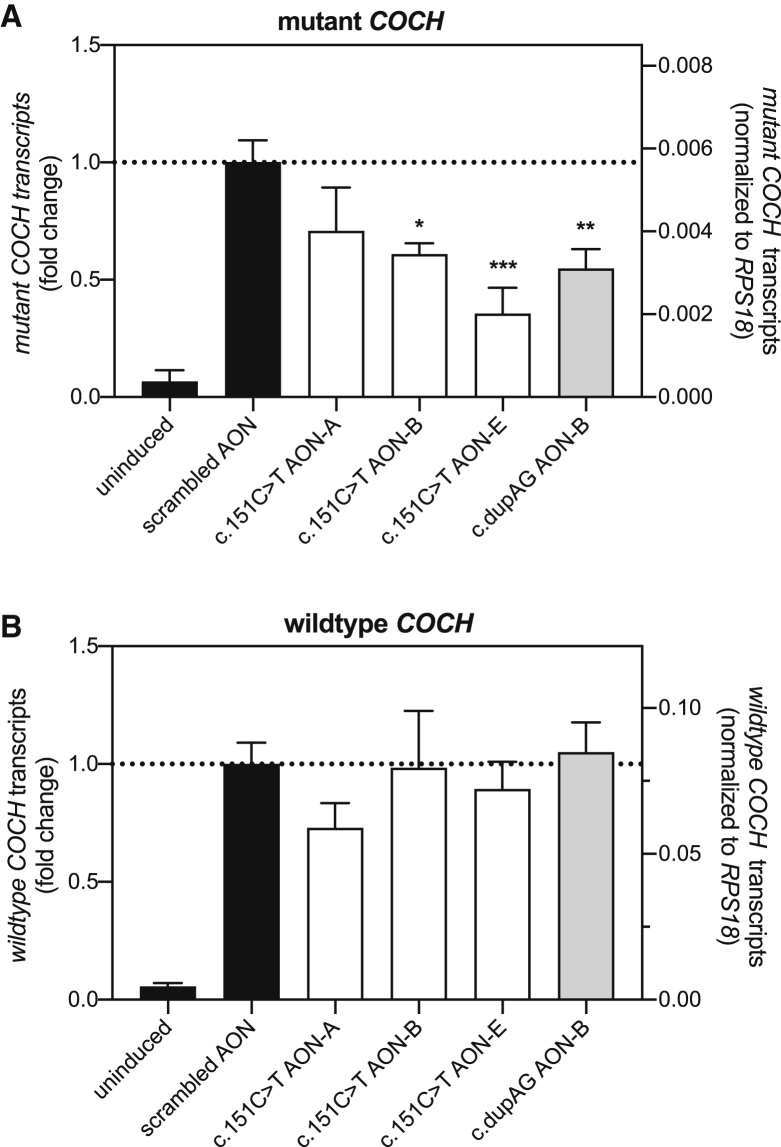
Comparison of AON efficiency in mutant and wild-type *COCH*-minigene-expressing cells (A and B) AONs directed against the c.151C>T mutation or the c.436+368_436+369dupAG (dupAG) variant were transfected in stable transgenic cell lines expressing (A) a mutant *COCH* minigene, and (B) a wild-type *COCH* minigene. AONs were transfected at a dose that results in a final concentration of 100 nM in the culture medium, and their effect on *COCH* transcript levels was investigated 24 h post transfection. (A) As shown previously, c.151C>T AON-B and AON-E, and dupAG AON-B, were able to induce a significant decrease in the mutant *COCH* transcript level. (B) None of the AONs resulted in a significant decrease in wild-type *COCH* transcript levels in transgenic cells expressing the wild-type *COCH* minigene. While c.151C>T AON-A results in a decrease in wild-type *COCH* transcript levels, the observed decrease is not statistically significant (p = 0.14, Tukey’s multiple comparison test). All AONs used here consisted of a gapmer composition. Mutant and wild-type *COCH* transcript levels are normalized for the expression of *RPS18* and plotted as mean ± SD of 3 replicates. The left axis shows the fold change compared to scrambled AON-transfected cells; the right axis shows the *RPS18* normalized transcript levels. ∗p < 0.05, ∗∗p < 0.01, ∗∗∗p < 0.001, one-way ANOVA with Tukey’s post-test.

We next investigated the ability of these four AONs in discriminating between mutant and wild-type *COCH* transcripts (Figure 4). We chose to compare the AONs at a concentration of 100 nM, as three out of the four AONs were able to significantly reduce mutant *COCH* transcript levels at this concentration. As observed previously, transfection of mutant *COCH* minigene cells with c.151C>T AON-B, c.151C>T AON-E, and dupAG AON-B significantly decreased mutant *COCH* transcript levels as compared to a scrambled control AON (Figure 4A). None of the four AONs induced a significant decrease of wild-type *COCH* transcripts when transfected in wild-type *COCH*-expressing transgenic cells, although we did observe a marked decrease in both mutant and wild-type *COCH* transcript levels resulting from the transfection of c.151C>T AON-A (Figure 4B). Likely, the correction for multiple comparisons explains the lack of a significant difference between c.151C>T AON-A-treated and scrambled AON-treated wild-type *COCH* minigene cells.

**Figure 5:**
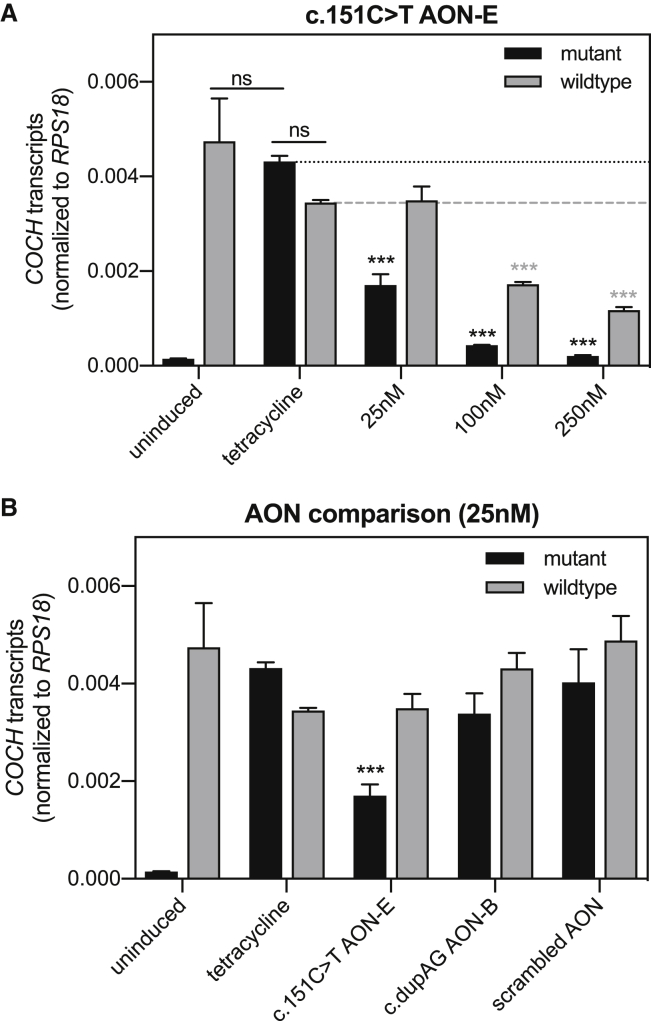
Investigation of allele-specificity in cells with equal levels of mutant and wild-type *COCH* transcription Mutant *COCH*-minigene-expressing cells from a clone with comparable expression levels of endogenous wild-type *COCH* were transfected with AONs. (A) Transfection of cells with c.151C>T AON-E results in a dose-depended decrease in mutant *COCH* transcript levels. Allele-specific reduction of mutant *COCH* transcript levels was observed at 25 nM but not at 100 and 250 nM. (B) Mutant and wild-type *COCH* transcript levels in cells treated with 25 nM c.151C>T AON-E, dupAG AON-B, or scrambled control AON. Mutant and wild-type *COCH* transcript levels are normalized for the expression of *RPS18* plotted as mean ± SD or 3 replicates. ∗∗∗p < 0.001, two-way ANOVA with Tukey’s multiple-comparison post-test conducted for possible comparisons. Outcome for relevant comparisons is shown; asterisks indicate the p value compared to tetracycline-treated cells.

While both cell lines display the same tetracycline-induced 18-fold increase in *COCH* minigene expression (Figure 4, left y axis), the expression levels relative to housekeeping gene *RPS18* differ between clones (Figure 4, right y axis). The higher normalized expression of wild-type *COCH* minigene transcripts as compared to mutant *COCH* minigene transcripts may lead to an overestimation of allele specificity of the AONs. Furthermore, the lack of mutant *COCH* transcripts in these cells poorly resembles the situation in patients. To better determine allele specificity, we searched for a mutant *COCH* minigene clone with similar expression levels of tetracycline-induced mutant *COCH* minigene and endogenous (wild-type) *COCH*. Subjecting these cells to three different AON concentrations revealed that c.151C>T AON-E can induce a significant and allele-specific reduction in mutant *COCH* transcript level at 25 nM (Figure 5A) (p < 0.0001, Tukey’s multiple comparison test). At 100 nM and 250 nM, c.151C>T AON-E induces a concomitant decrease in endogenous wild-type *COCH* levels. Nevertheless, the 4- to 6-fold lower levels of mutant *COCH* transcripts as compared to wild-type *COCH* transcripts indicate that c.151C>T AON-E has a stronger affinity for the mutant transcript. In the same cell model, transfection of 25 nM of dupAG AON-B did not result in significant differences in mutant and wild-type *COCH* transcript levels (Figure 5B). A scrambled control AON was included in this experiment to confirm that the observed effect is specific to the AON sequence. Transfection of the cells with the scrambled AON mildly increases endogenous wild-type *COCH* expression but not the expression of the tetracycline-induced mutant *COCH* minigene. Although compared to the delivery of scrambled AON, c.151C>T AON-E does reduce the levels of wild-type *COCH* transcripts with 30% (p = 0.14, Tukey’s multiple comparison test), the comparison with untransfected cells expressing both mutant and wild-type *COCH* transcripts is most relevant from a therapeutic point of view.

## Discussion

The c.151C>T founder mutation in *COCH* is estimated to be one of the most prevalent causes of dominantly inherited, adult-onset hearing loss and vestibular dysfunction in Northwest Europe, affecting >1,000 individuals in the Dutch/Belgian population. In this work, we present 11 intronic variants in *cis* with the c.151C>T mutation and show that these variants can be exploited for the development of a mutant-allele-specific therapy using RNase H1-dependent AONs. We identified a highly effective AON, directed against the c.151C>T mutation, as the most promising candidate for further preclinical development.

The ability of AONs to specifically target mutant transcripts for degradation is of key importance for the development of an AON-based therapy for dominantly inherited disorders with a dominant-negative or gain-of-function disease mechanism such as DFNA9. The therapeutic strategy must be potent enough to prevent the synthesis of proteins from the mutant allele but allow sufficient protein synthesis from the wild-type allele for normal inner ear function. For any antisense-based approach, discrimination between alleles based on a single-nucleotide difference presents as a potential pitfall in terms of concomitant downregulation of the wild-type allele.^22^, ^23^, ^24^ Recently published AONs directed against a mutation in *NR2E3*, causative for dominantly inherited retinitis pigmentosa, also significantly reduced the wild-type transcript and protein levels.^21^ In contrast, for Huntington’s disease (*HTT* gene), also resulting from a non-haploinsufficiency disease mechanism, the use of gapmer AONs to target a SNP specific to the mutant allele emerged as a promising therapeutic strategy *in vitro* and *in vivo*.^15^ As nearly all cases of DFNA9 are caused by single-nucleotide changes,^25^ we explored the presence of mutant-allele-specific variants that can serve as additional targets to develop a therapeutic strategy for the most frequently occurring DFNA9 mutation, c.151C>T. Here, we employed SMRT sequencing^26^ to sequence the complete mutant *COCH* haplotype using three overlapping PCR amplicons. With average polymerase read lengths of up to 30 kb, the SMRT sequencing platform presents a powerful tool to identify genetic variants on the mutant allele and offers a major advantage over the manual genotyping of family trios, as done for example to identify the target SNPs in the *HTT* gene.^27^

The c.151C>T *COCH* allele contains a remarkably high number of SNPs with a relatively low allele frequency (∼5%) in the non-Finnish European population according to the gnomAD database (v.2.1.1).^28^ As the c.151C>T founder mutation arose on a relatively uncommon haplotype, we estimate that less than 5% of DFNA9 patients are homozygous for these SNPs. Therefore, approximately 95% of DFNA9 patients with the c.151C>T mutation can theoretically be treated with AONs directed against one of these mutant-allele-specific variants. In comparison, it was reported that targeting one of three relatively frequent SNPs can provide a treatment for approximately 85% of patients suffering from Huntington’s disease.^27^ In contrast to the identified mutant-allele-specific SNPs in *HTT*, all of the identified variants in *COCH* map to the introns. As such, the identified mutant-allele-specific variants in *COCH* are only amenable to AON-mediated pre-mRNA degradation by the RNase H1 enzyme and not to mRNA interference (RNAi).^29^, ^30^, ^31^

We designed AONs to specifically target mutant *COCH* transcripts for RNase H1 degradation. In addition to targeting the DFNA9-associated mutation c.151C>T, we opted to target the 2 bp duplication c.436+368_436+369dupAG in *cis* with the DFNA9 mutation. *In silico* analysis of thermodynamic AON properties indicated that AONs directed against the dupAG variant possess a larger difference in binding affinity between the mutant and the wild-type transcript as compared to AONs directed against the c.151C>T mutation (Table S1). The on-target efficacy of AONs was investigated in stable transgenic cells that express a mutant *COCH* minigene under control of a tetracycline-dependent promotor. A similar cell model was previously used to investigate the kinetics of RNase H1-dependent antisense oligonucleotide induced degradation^13^ and offers a suitable alternative to the patient-specific fibroblast and lymphoblastoid cell lines that hardly express *COCH*. We opted to investigate the effect of AONs under continuous activation of *COCH* transcription, which best resembles the situation in the cochlea, where constant *COCH* expression amounts to synthesis of one of the most abundant proteins in the entire organ.^1^^,^^6^ The gapmer configuration of c.151C>T AON-E was the most effective of all the designed AON molecules and at the highest dose resulted in a decrease of mutant *COCH* transcripts to <15% of the number of transcripts in cells treated with a scrambled control AON. The effects of AONs directed against the c.436+368_436+369dupAG (dupAG) variant were overall lower as compared to the c.151C>T AONs. This could result from small differences in biochemical properties. The target region of the dupAG variant is more AT rich as compared to the sequence surrounding the c.151C>T variant. The calculated free energy of on-target AON binding was indeed lower for AONs directed against the dupAG variant as compared to AONs directed against the c.151C>T mutation. The fact that these AONs are directed against an intronic variant, which is only present in unspliced nuclear pre-mRNA, could also contribute to the lower on-target efficiency.

In terms of specificity for the mutant *COCH* allele, we anticipated an advantage for AONs directed against the dupAG variant. When transfected in wild-type *COCH* minigene-expressing cells, both c.151C>T AON-E and dupAG AON-B did not reduce the levels of wild-type *COCH*. In view of the higher expression of wild-type *COCH* as compared to mutant *COCH*, we concluded that this approach could possibly overestimate the allele specificity. We searched for a mutant *COCH* minigene clone with similar (tetracycline induced) mutant *COCH* expression as endogenous wild-type *COCH* expression. Transfection of c.151C>T AON-E in these cells revealed that allele specificity strongly depends on the amount of AON that is delivered to the cells. At 25 nM, the levels of mutant *COCH* transcripts were decreased to 40% of the levels of mutant *COCH* in control cells, without reducing the number of wild-type *COCH* transcripts. Upon increasing the concentration, c.151C>T AON-E also reduced the number of wild-type transcripts, albeit less compared to the reduction in mutant transcript levels. While dupAG AON-B also did not reduce wild-type *COCH* levels at 25 nM, the effect on mutant *COCH* transcripts was mild and therefore currently of little relevance for future clinical applications, especially since transfections with increasing doses did not further decrease the levels of mutant *COCH* transcripts in the initial dose-response study. While the on-target efficiency of AONs directed against the dupAG variant can be improved by, for example, increasing the length of the AON or introducing chemically modified nucleotides that enhance binding affinity, we see little reason for this. With higher efficiency and allele specificity at 25 nM, c.151C>T AON-E is a better candidate for clinical applications, especially since the use of AONs directed against the pathogenic mutations, unlike those directed against allele-specific variant, is not constrained by a small percentage of individuals who are homozygous for the target variant.

While c.151C>T AON-E could also benefit in terms of allele specificity from chemical modifications, decreasing AON length and or shortening the gap region, these alternations are likely to decrease cleavage efficiency as well. This raises the question of how much reduction in mutant *COCH* transcripts is required to achieve a clinically meaningful effect. Lacking an animal model to perform these experiments, we can currently only discuss the different factors that will determine the therapeutic effect. First of all, our data suggest that allele specificity strongly depends on the expression levels of mutant and wild-type *COCH*. Therefore, the AON dose is best titrated *in vivo*. In addition, we should realize that the levels of mutant and wild-type *COCH* are not necessarily identical, as allelic variation in gene expression is quite common in the human genome.^32^ Second, we do not know yet how much knockdown of mutant *COCH* is required to alleviate the patient’s phenotype. The age of onset of symptoms in DFNA9, usually between the 3^rd^ and 5^th^ decade of life for the c.151C>T mutation,^2^^,^^33^^,^^34^ indicates that the cochlea can cope with the burden of mutant cochlin proteins for many decades. It has furthermore been shown that otic fibrocytes, the main cell type producing cochlin, display some capacity for self-renewal.^35^ In the most optimal situation, AONs might be able to remove the burden of mutant cochlin proteins to an extent that allows for fibrocyte renewal and thereby possibly improved auditory and vestibular function. We speculate that a >50% reduction of mutant transcript levels, such as achieved upon the transfection of 25 nM of c.151C>T AON-E, could already be sufficient to halt or delay the disease progression when treatments are started at an early stage. The third factor is the extent to which a reduction in wild-type *COCH* transcripts is tolerated. We know from animal models and carriers of loss-of-function mutations that the number of wild-type transcripts from a single allele is sufficient for normal inner ear function. While difficult to put a number on the minimal number of transcripts needed, studies on splice modulation therapy for *USH1C*-associated hearing loss indicate that 20% of functional *USH1C* transcripts is sufficient for inner ear function.^36^ Along these lines, the 80% reduction of mutant transcripts and 50% reduction of wild-type transcripts that we observe upon the transfection of 100 nM c.151C>T AON-E could potentially yield good therapeutic outcomes.

The transient effect of AONs is both an advantage and a potential limitation for future clinical applications. It lowers the risk of sustained adverse or off-target effects that could accompany genome-editing techniques, but it also implies that a repeated delivery is likely to be required to achieve maximum efficacy. AON-based splice-modulation therapy for hearing impairment in Usher syndrome type 1c is extensively investigated in the fetal and post-natal cochlea.^36^^,^^37^ In contrast to *USH1C*-associated hearing impairment, the adult age of onset of DFNA9 leaves ample opportunity for therapeutic intervention in adults before the onset of the initial symptoms. Delprat et al.^38^ previously reported the use of phosphorothioate oligonucleotide-mediated knockdown in the adult rodent cochlea to investigate the role of the otospiralin protein in the inner ear protein. In this study, they placed pieces of gel foam loaded with AONs on the round window membrane (RWM) of rats and observed the effects of otospiralin knockdown already 2 days later.^38^ Otospiralin and cochlin are both expressed by the otic fibrocytes, which indicates that cellular uptake of AONs is unlikely to be a limiting factor for future AON-based DFNA9 therapy. Although many advancements in cochlear drug delivery have been made since,^39^, ^40^, ^41^ a huge gap in knowledge remains in terms of safety, stability, and biodistribution of gapmer AONs in the (adult) human cochlea. Further investigation into the feasibly of RWM diffusion as a potential delivery method for AON-based therapy in patients is also warranted, as the gapmer composition of AONs may affect diffusion properties, and the thickness of the human RWM and larger size of the cochlea are likely to affect the biodistribution.

In conclusion, this study shows that AONs can be engineered to target the c.151C>T mutant *COCH* transcript for degradation. The identified intronic, mutant-allele-specific variants present interesting alternative targets to design allele-specific AONs. However, the AONs directed against the intronic variant in *COCH* that were investigated here require further optimization of knockdown efficiency and specificity before continued preclinical development is warranted. The best-performing AON directed against the pathogenic mutation is able to reduce mutant *COCH* transcripts by 60% without affecting levels of wild-type transcripts. While increasing AON concentration further increased the reduction in mutant *COCH* transcripts, the levels of wild-type transcripts no longer remain unaffected. Further pre-clinical studies in animal models of DFNA9 are needed to assess the clinically relevant reduction in mutant *COCH* transcript levels and to what extent a decrease in wild-type transcripts is tolerated. The rapidly evolving procedures for repeated drug delivery to the cochlea render the application of AONs for the treatment of inherited hearing impairment an increasingly feasible strategy.

## Materials and methods

### SMRT sequencing of *COCH* haplotypes

This study was approved by the medical ethics committee of the Radboud University Medical Center in Nijmegen, the Netherlands and was carried out according to the Declaration of Helsinki. Experiments were conducted at the Radboud University Medical Center in Nijmegen, the Netherlands. Written informed consent was obtained from all participants. DNA samples of three seemingly unrelated DFNA9 patients carrying the c.151C>T mutation in *COCH* were selected for SMRT sequencing (Pacific Biosciences, Menlo Park, CA, USA) to identify shared variants on the mutant allele. The *COCH* gene was amplified in three overlapping amplicons (Figure 1), in which known haplotype-specific variants were anticipated to be present to aid assembly. Fragments were amplified with primers 5′-GAAGTTCGGTTCTCAGGCC-3′ and 5′-TGCCATCGTCATACAAAAGG-3′ (fragment 1), 5′-CAAAATCTGGAATGGTATGGAAG-3′ and 5′-GATCAAATGCAGACCTAGCC-3′ (fragment 2), and 5′-TCCCCTGCAGTACTTTTTGTC-3′ and 5′-GTAAGCCAGCTTACAATAACTC-3′ (fragment 3), using Q5 polymerase (New England Biolabs, Ipswich, MA, USA) according to manufacturer’s instructions. Amplicons were pooled per sample, and library preparation was done according to protocol Procedure and Checklist—Preparing SMRTbell Libraries using PacBio Barcoded Adapters for Multiplex SMRT Sequencing (Pacific Biosciences, #100-538-700-02). Generation of polymerase-bound SMRTbell complexes was performed using the Sample Setup option in SMRTLink 6.0 (Pacific Biosciences), and sequencing was performed on a Sequel I systems (Pacific Biosciences). Following the run, generation of circular consensus reads (CCS) and mapping of these reads was performed using SMRTLink 6.0. Bam files were loaded into the Sequence Pilot software (JSI Medical Systems) to perform variant calling. The variants were subsequently filtered to excluded homopolymers and homozygous variants. The identified variants with a low population frequency (<10%) were considered as potential therapeutic targets and validated using targeted Sanger sequencing. Segregation analysis in two branches of large Dutch DNFA9 families (W02-006 and W00-330) was used to confirm the presence of the identified variants on the mutant haplotype. Primers used in the segregation analyses are listed in Table S2.

### AONs

AONs were designed using previously published criteria for splice-modulating AONs.^17^^,^^18^ In summary, the sequences surrounding the c.151C>T and c.436+368_436+369dupAG variants on the mutant *COCH* allele were analyzed *in silico* for AON accessibility. The thermodynamic properties of every possible 20-mer antisense oligonucleotide were analyzed *in silico* for AON-AON duplex formation, the formation of AON-target mRNA duplexes, and the formation of AON-wild-type mRNA duplexes using the RNAstructure webserver.^42^ The uniqueness of the AON target sequences was determined by BLAST analysis. The seven most optimal AONs were purchased from Eurogentec (Liège, Belgium) and dissolved in phosphate-buffered saline (PBS) before use. As a non-binding control, an AON with a scrambled nucleotide sequence was also acquired. Sequences and AON chemistry are presented in Table S1.

### Generation of transgenic *COCH* minigene cell lines

The genomic region of wild-type and c.151C>T mutant *COCH* exons 1 to 7 (transcript variant 1; NM_001135058.1), including the haplotype-specific variants, was amplified from the translation initiation site to the splice donor site of exon 7 using primers 5′-ATGTCCGCAGCCTGGATC-3′ and 5′-GGCTTGAACAAGGCCCACA-3′. The mutant and wild-type amplicons were subsequently cloned into the pgLAP1 vector (Addgene plasmid #19702) using Gateway cloning technology (Invitrogen, Carlsbad, CA, USA). Upon sequence validation, *COCH*-containing pgLAP1 vectors were co-transfected with pOGG44 (#V600520, Invitrogen), encoding Flp-Recombinase, in FLp-in T-REx 293 cells (#R78007, Invitrogen) using polyethylenimine. Cells in which the *COCH* sequence was stably integrated were selected for using DMEM-AQ medium (Sigma Aldrich, Saint Louis, MO, USA) supplemented with 10% fetal calf serum, 1% penicillin/streptomycin, sodium pyruvate, 10 μg/mL blasticidin, and 100 μg/mL hygromycin. Individual hygromycin-resistant clones were expanded and subsequently tested for the induction of *COCH* transcription by tetracycline using an allele-specific TaqMan assay. Correct splicing of the *COCH* minigenes was assessed using a forward primer on exon 1 (5′-TCCGCAGCCTGGATCCCGG-3′) and reverse primer on exon 7 (5′-GGCTTGAACAAGGCCCACA-3′).

### Delivery of RNase H1-dependent AONs

Wild-type and mutant *COCH*-expressing FLp-in T-REx 293 cells were cultured in DMEM-AQ medium (Sigma Aldrich, Saint Louis, MO, USA) supplemented with 10% fetal calf serum, 1% penicillin/streptomycin, sodium pyruvate, 10 μg/mL blasticidin, and 100 μg/mL hygromycin. For AON treatments, cells were seeded in 12-well or 24-well plates at ∼50% confluency. Next day, *COCH* transcription was activated through the administration of 0.25 μg/mL tetracycline (#T7660, Sigma Aldrich). Twenty hours after induction, cells were transfected with AONs using Lipofectamine 2000 (Invitrogen) according to manufacturer’s instructions, using a 1:2 ratio of AON (in μg) and lipofectamine reagent (in μL). AON doses are calculated as final concentration in the culture medium. Cells were collected for transcript analysis 24 h after AON delivery.

### RNA extraction and cDNA synthesis

Total RNA was extracted from cells using the Nucleospin RNA mini kit (#740955, Machery-Nagel) according to manufacturer’s instructions. First-strand cDNA was generated using iScript cDNA synthesis reagents (Bio-Rad, Hercules, CA, USA) using a fixed amount of RNA input (250 ng) in a 10 μL reaction volume. The obtained cDNA was diluted four times and used for transcript analysis.

### Analysis of *COCH* transcript levels

Diluted cDNA (4 μL) was used as input in an allele-specific TaqMan assay using primers 5′-GGACATCAGGAAAGAGAAAGCAGAT-3′ and 5′-CCCATACACAGAGAATTCCTCAAGAG-3′, a wild-type allele-specific VIC-labeled probe 5′-CCCCCTGGGCAGAG-3′ and a mutant allele-specific FAM-labeled probe 5′-CCCCCTGAGCAGAG-3′. Expression of *RPS18* was analyzed with GoTaq (#A6002, Promega), using primers 5′-ATACAGCCAGGTCCTAGCCA-3′ and 5′-AAGTGACGCAGCCCTCTATG-3′. Abundance of mutant and wild-type *COCH* transcripts was calculated relative to the expression of the housekeeping gene *RPS18*.

## Supplemental information

Document S1. Figures S1 and S2 and Tables S1 and S2

Document S2. Article plus supplemental information
