# Supplementary material for: AON-based degradation of c.151C>T mutant COCH transcripts associated with dominantly inherited hearing impairment DFNA9
Source: Mol Ther Nucleic Acids. 2021 Mar 1;24:274–83. doi: 10.1016/j.omtn.2021.02.033 (PMC7985667; doi:10.1016/j.omtn.2021.02.033)
Supplement: Document S1. Figures S1 and S2 and Tables S1 and S2 [file mmc1.pdf]

OMTN, Volume *24*

Supplemental information

AON-based degradation of c.151C>T mutant *COCH* transcripts associated with dominantly inherited hearing impairment DFNA9

Erik de Vrieze, Jorge Cañas Martín, Jolien Peijnenborg, Aniek Martens, Jaap Oostrik, Simone van den Heuvel, Kornelia Neveling, Ronald Pennings, Hannie Kremer, and Erwin van Wijk

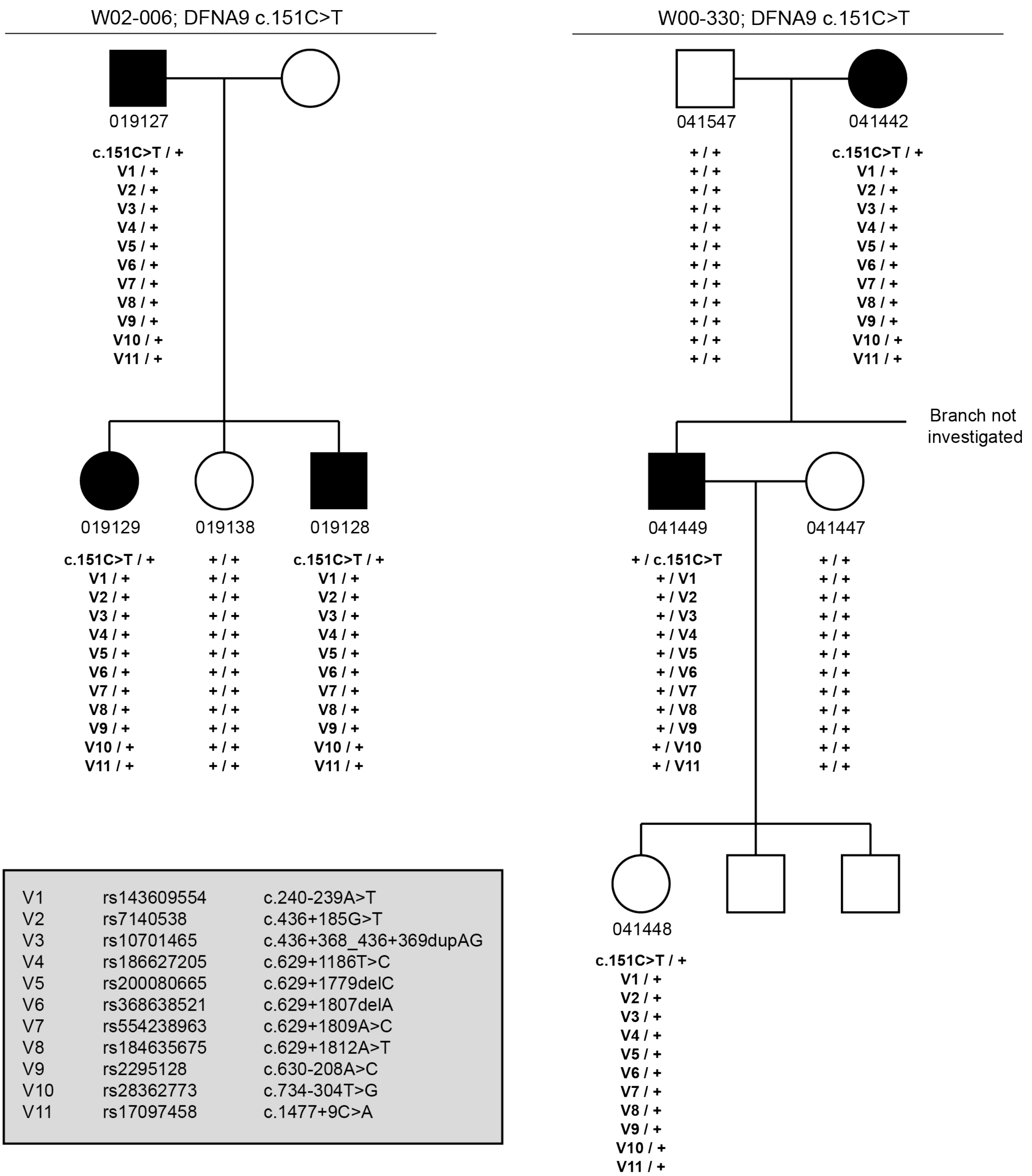

V1	rs143609554	c.240-239A>T
V2	rs7140538	c.436+185G>T
V3	rs10701465	c.436+368_436+369dupAG
V4	rs186627205	c.629+1186T>C
V5	rs200080665	c.629+1779delC
V6	rs368638521	c.629+1807delA
V7	rs554238963	c.629+1809A>C
V8	rs184635675	c.629+1812A>T
V9	rs2295128	c.630-208A>C
V10	rs28362773	c.734-304T>G
V11	rs17097458	c.1477+9C>A

Figure S1. Segregation analysis of haplotype-specific variants. Small branches from the pedigrees of two large Dutch DFNA9 families (W02-006 and W00-330) were investigated to confirm co-segregation of the haplotype-specific variants with the c.151C>T mutation. Numbers below each individual depict the internal identifier of the DNA samples. Individual 041448 was not clinically affected at the time of sample collection. V1-V10: *COCH* variants (see grey box); +: wildtype; square: male; circle: female; open symbol: clinically unaffected; closed symbol: clinically affected.

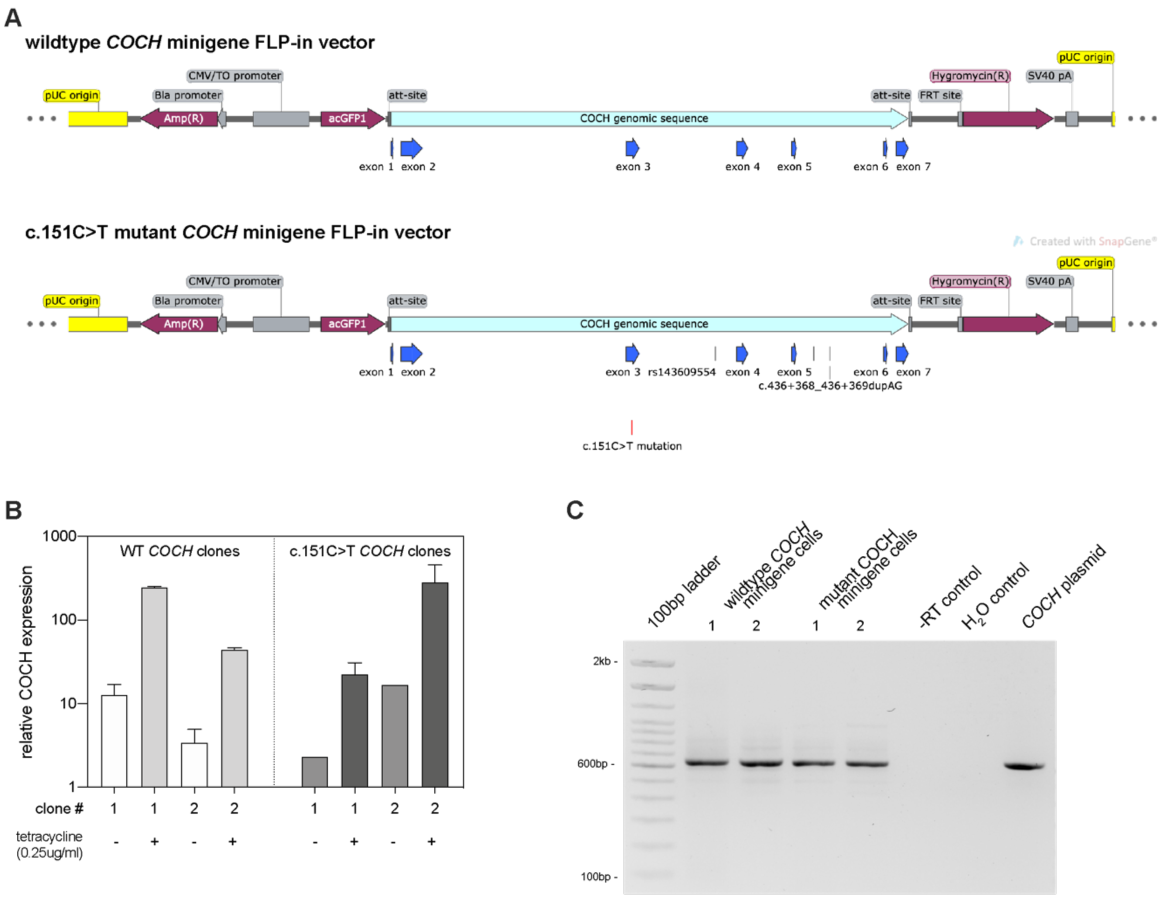

Figure S2. Inducible *COCH* minigene T-REx 293T cells. A) schematic overview of the wildtype and mutant COCH vectors that were used to establish the COCH minigene T-REx 293T cells. **B)** Measurement of *COCH* expression upon overnight induction with tetracycline. Two clones of wildtype *COCH* minigene-expressing transgenic cells, and two clones of mutant *COCH* minigene-expressing transgenic cells were investigated. Wildtype clone 2, and mutant clone 1 were selected for experiments based on the relatively similar levels of *COCH* expression upon tetracycline treatment. Note that uninduced cells always show a certain level of background *COCH* expression. As the Taqman™ probe for the mutant *COCH* transcript is highly specific, it appears that the transcriptional activity of the tetracycline promotor is not completely off in uninduced cells. Data shown as mean ± SD. **C)** RT-PCR analysis of *COCH* transcripts in tetracycline-treated mutant and wildtype *COCH* minigene-expressing cells. For each cell line, two replicate samples are shown. Sanger sequencing of the amplicons confirmed correct splicing of the minigene *COCH* transcripts. The positive control is a plasmid containing the coding sequence of *COCH* that was amplified from fetal cochlear cDNA.

Table S1: AON sequences and chemical and thermodynamic properties. Phosphorothioate links in the AON sequences are indicated by the asterisks between bases. Variant c.436+368_436+369dupAG is abbreviated in the table as dupAG. The 2'-O-methyl-RNA bases are placed between brackets. The allele-discriminating variants are indicated by bold underlined fonts. Secondary structure and free energy predictions are done with the RNAstructure webserver (http://rna.urmc.rochester.edu/RNAstructureWeb/). The RNAstructure webserver can not take the chemical modification or gapmer composition into account. Therefore, gapmers were analysed as both DNA and RNA molecules. # AONs were investigated in gapmer chemistry and complete PS-DNA chemistry

			**AON secondary structure free energy (kcal/mol)**		**self dimerisation (kcal/mol)**		**free energy of bimolecular structure (kcal/mol)**		**Difference in free energy between mutant and wildtype bimolecular structure (kcal/mol)**
**AON name**		**AON sequence**	*as DNA*	*as RNA*	*as DNA*	*as RNA*	*mutant COCH*	*wildtype COCH*	*delta*
c.151C>T	AON-A	(C*C*C*)T*G***A***G*C*A*G*A*G*G*A*C*A*T*(C*U*G)	-1,9	-3,0	-7,1	-13,4	-41,0	-36,2	4,8
	AON-B	(C*U*G*)**A***G*C*A*G*A*G*G*A*C*A*T*C*T*(G*C*U)#	-5,3	-7,5	-12,6	-18,0	-39,4	-34,6	4,8
	AON-C	(C*C*C*C*)T*G***A***G*C*A*G*A*G*G*A*C*(A*U*C*U)	1,4	-3,0	-7,1	-14,2	-41,7	-36,9	4,8
	AON-D	T*G*A*G*C*A*G*A*G*G*A*C*A*T*C*T*G*C*T*T	-5,3	-	-12,6	-	-37,8	-33,0	4,8
	AON-E	(A*G*C*C*)C*C*C*T*G***A***G*C*A*G*A*G*(G*A*C*A)#	1,4	-3,0	-6,3	-14,6	-44,5	-39,7	4,8
	AON-F	(C*C*U*G*)**A***G*C*A*G*A*G*G*A*C*A*T*(C*U*G*C)	-4,4	-5,7	-9,9	-14,4	-41,1	-36,3	4,8
	AON-G	G*C*A*G*C*C*C*C*C*T*G***A***G*C*A*G*A*G*G*A	-0,8	-	-7,2	-	-46,1	-41,3	4,8
dupAG	AON-A	(U*C*A*U*)A*G*C*T*A*G*A*C***C*T*C*T***(G*U*C*U)	-1,6	-1,6	-8,8	-9,1	-36,1	-27,0	9,1
	AON-B	(A*U*A*G*)C*T*A*G*A*C***C*T*C*T***G*T*(C*U*A*A)#	-2,2	-3,7	-8,8	-10,6	-33,9	-24,8	9,1
	AON-C	(A*U*C*A*)T*A*G*C*T*A*G*A*C***C*T*C*(U***G*U*C)	-0,8	1,4	-8,8	-8,9	-35,6	-26,6	9,0
	AON-D	C*A*U*C*A*T*A*G*C*T*A*G*A*C***C*T*C*U***G*U	1,5	-	-7,8	-	-35,3	-27,5	7,8
	AON-E	A*G*C*T*A*G*A*C***C*T*C*T***G*T*C*T*A*A*A*A	-2,2	-	-8,8	-	-33,3	-24,2	9,1
	AON-F	(U*A*G*C*)T*A*G*A*C***C*T*C*T***G*T*C*(U*A*A*A)	-2,2	-3,7	-8,8	-10,6	-33,7	-24,5	9,2
	AON-G	C*A*T*A*G*C*T*A*G*A*C***C*T*C*T***G*T*C*T*A	-2,1	-	-8,8	-	-35,5	-26,9	8,6
scrambled AON		(G*C*T*A*)T*C*G*A*T*T*A*C*A*C*T*A*(T*C*G*A)	-2,3	-3,6	-7,4	-9,4			

Table S2 - primer list for segregation analysis of identified allele-specific variants

variant	forward primer	reverse primer	sequencing primer
c.151C>T	CACTGTAGTCTCCCCACCAC	CAGATGGGTAAAGCAGGAAAG	
c.240-239A>T	TCACACCTGTAATCCCACCA	CCACACTTTTTCAGGGCATC	
c.436+185G>T	ACAAGCAGTGTCCACAGCAC	CTGAACTTTGGGAGGCTGAA	CCCGGCACAGCATTTGGAAG
c.436+368_436+369dupAG			
c.629+1186T>C	AGACCATCCTGGCTAACACG	TTTTCAAGCTTTCTATAATGAGCA	
c.629+1779delC	CCTGGCCCCTTCAGTATTTT	CTGAGCAGCTGGCACTACAG	
c.629+1807delA			
c.629+1809A>C			
c.629+1812A>T			
c.630-208A>C	GCTGTGTTTCATCAGGCAAA	TTGGAATTACCCCCTCTGAA	
c.734-304T>G	GAATGCAGATGTGGCAGAAA	GATGCATCAGCTGGGAAAGT	
c.1477+9C>A	TGGTGGAACAGCTACTGGTG	TGGTGGAACAGCTACTGGTG	
